# Does improving the skills of researchers and decision-makers in health policy and systems research lead to enhanced evidence-based decision making in Nigeria?—A short term evaluation

**DOI:** 10.1371/journal.pone.0238365

**Published:** 2020-09-03

**Authors:** Obinna Onwujekwe, Enyi Etiaba, Chinyere Mbachu, Ifeyinwa Arize, Chikezie Nwankwor, Uchenna Ezenwaka, Chinyere Okeke, Nkoli Ezumah, Benjamin Uzochukwu

**Affiliations:** 1 Department of Health Administration and Management, University of Nigeria, Enugu Campus, Enugu Town, Nigeria; 2 Health Policy Research Group, College of Medicine, University of Nigeria, Enugu Campus, Enugu Town, Nigeria; 3 Department of Community Medicine, University of Nigeria, Enugu Campus, Enugu Town, Nigeria; University of British Columbia, CANADA

## Abstract

**Introduction:**

Health care decision makers require capacity to demand and use research evidence for effective decision making. Capacity to undertake health policy and systems research (HPSR) and teaching is low in developing countries. Strengthening the capacity of producers and users of research is a more sustainable strategy for developing the field of HPSR in Africa, than relying on training in high-income countries.

**Methods:**

Data were collected from 118 participants who had received the capacity building, using a pre-tested questionnaire. Respondents included health research scientists from institutions (producers) and decision makers (users) in the public health sector, in Anambra and Enugu states, southeast Nigeria. Data were collected on participants’ progress with proposed group activities in their short- term goals; effects of these activities on evidence-informed decision making and constraints to implementing activities. Univariate analysis was done using SPSS version 16.

**Findings:**

All prioritised activities were carried out. However, responses were low. Highest response for an activity amongst producers was 39.1%, and 44.4% for users. Some of the activities implemented positively influenced changes in practice; like modification of existing policies and programme plans. There was a wide range of responses between producers of evidence (0.0–39.1%) and users (2.7–44.4%) across both study states. Lack of authority to implement activities was the major constraint (42-9-100.0% across activities), followed by financial constraints (70.6%).

**Conclusion:**

Capacity building intervention improved skills of a critical mass of research scientists, policymakers and practitioners, towards evidence-based decision making. Participants committed to undertake proposed activities but faced a number of constraints. These need to be addressed, especially the decision space and authority, improving funding to implement activities that influence Getting Research into Policy & Practice (GRIPP). Being at different stages of planning and implementing proposed activities; participants require continuous technical and financial support to successfully implement activities and engage meaningfully within and across professional boundaries and roles, in order to achieve short-, medium- and long- term goals.

## Introduction

Sustainable research capacity and evidence-informed policy making require health research professionals and policymakers with in-depth scientific expertise and complementary skills that enable them to conduct independent infectious disease research with global appeal but relevant to the health priorities of their country. Hence, Health Policy and Systems Research (HPSR) is recognized as critical in the success of health interventions and programmes.

Historically, the field of HPSR started to emerge a little over two decades ago. At the time, a number of challenges were identified; an agreed definition of the field, dominance of clinical and biomedical research and no demand for HPSR at the time, but a major factor and cross cutting issue was the limited capacity to undertake HPSR, and this was even more so in Low and Middle Income Countries (LMICs)[1]. Although substantial progress has been made since then, in terms of increasing HPSR studies and publications[2], capacity to undertake health policy and systems research and teaching is still low in developing countries[3–6]. Strengthening the capacity of producers and users of research has been argued to be a more sustainable strategy for developing the field of HPSR in Africa, than relying on training in high-income countries. [2, 7–10]

As both policymakers and communities increasingly demand better returns on investments in health, HPSR has the potential to enable health system interventions to achieve better value for money. To address this, producers and users of HPSR evidence need to be trained and empowered locally, to be more context useful. The long-term goal is to strengthen individual and institutional capacity to initiate and lead research activities in disease endemic countries, while developing national and international partnerships. HPSR is typically context-specific and to apply research evidence to policy, national-level capacity is needed [11, 12], and in addition, an understanding of the local policy making process [13–16].

There is a current need to build the capacity of HPSR in LMICs as this encompasses the processes of decision-making at all levels of the health system.[7, 17, 18] HPSR enables the identification of gaps in capacity, barriers to efficient functioning and effective performance of the health system and methods by which the existing resources can be optimally utilized.[7, 19]. The three levels of capacity in health systems research are: i) Individual capacity (skills in research, grant and report writing, communication of research findings etc.); ii) Organizational capacity (establishment of appropriate organizational incentives and rewards for engaging in research, library and information technology, financial systems for grant management, established career pathways, research leadership etc.) and iii) Environmental and Network Capacity (development of networks between different research organizations (both within countries and regionally or internationally), links to policy and decision makers within the health system, and established national systems for identifying priority HPSR needs and supporting such research)[20].

There is lack of demand for research and research findings, especially in HPSR for decision-making in LMICs [21, 22]. Some of the factors contributing to this lack of demand is the meagre appreciation of the value of research and its potential to contribute to policy development. It was also observed that an environment or culture conducive to health research is lacking in many LMICs [21].

In Nigeria, tertiary educational institutions such as universities are important to strengthening and sustaining capacity in HPSR. They, not only produce knowledge through research but are also mandated to teach the next generation of policy-makers, health professionals and researchers[3]. However, there is limited capacity amongst these groups due to the long-standing culture of not making research a priority and poor funding for research[23–25]. Research capacity encompasses not only the capacity to produce research, but also the capacity to demand and use it, so that research knowledge may contribute to improvements in health and health equity.

In recognition of the need to build capacity for HPSR that will lead to demand and use of HPSR for decision-making, especially for the control of endemic tropical disease in Nigeria, a 12-month implementation research project was undertaken to build the capacity of a critical mass of research scientists, policy/decision makers and practitioners who: (i) know that successful endemic disease control programmes rely on evidence-informed decision making, and that HPSR and economic evaluation are viable tools for producing research evidence; (ii) commit to undertake activities towards improving the use of research evidence in endemic disease control in their respective States; and (iii) are willing to form research and practice networks with other producers and users of evidence.

The ultimate goal of the project was to build the requisite capacity that can bridge the gap between researchers, practitioners and policymakers in the study areas, and hence facilitate and enhance getting research into policy & practice (GRIPP)[2, 26].

The paper provides new knowledge on how capacity for Health Policy and Systems Research and Analysis (HPSR+A) and GRIPP was built for researchers (producers of evidence) and programme managers (users of evidence) and the level to which they used this capacity in reviewing, re-designing programming, implementing and evaluating strategies and plans for the control of endemic diseases in two states in southeast Nigeria. Hence, it shows how the level of the skills that users and producers of evidence gained from a capacity development project was used by both the users and producers of evidence whose capacities were developed to improve HPSR evidence-based decision making and production of HPSR evidence in their organisations, and how these improve their activities for the control of endemic diseases in the two states. Using two states for the study was not for direct comparison of the outputs, but rather to illustrate the need for context-specificity in planning and undertaking activities, given varying contexts. The paper therefore contributes the experiences of the study context to the growing field of HPSR and knowledge translation.

## Methods

### Study area and setting

This capacity building project was implemented in Anambra and Enugu states. Both states are located in the south east geo-political zone of Nigeria. Anambra and Enugu states have an estimated population of 4.2 million and 3.3 million respectively, based on an annual growth rate of 2.8% [27, 28]. The health care system aligns with the country’s three tier system of government, such that the federal level is responsible for teriary care through the tertiary health institutions, the state level takes care of secondary care through general hospitals and cottage hospitals while the local government tier is in charge of primary health care. However, some states also own tertiary institutions, in such cases this is overseen by the state[29]. Anambra and Enugu states each have one federal owned and one state owned tertiary institution and these four institutions are central to strengthening HPSR+A Capacity in the country. The College of Medicine University of Nigeria, Enugu campus (COMUNEC) has striven to bring HPSR to the fore in the country by collaborating with policy makers and international partners and have made some internal progress, especially in GRIPP [30].

As with other regions in Nigeria, prevalence of malaria is high, and the disease burden is highest among pregnant women and children under five years of age. Similarly, prevalence of Neglected Tropical Diseases (NTDs) is high in the region[27, 31]. At the time of the study, both states were receiving funding support through Saving One Million Lives (SOML) programme to expand access to essential primary health care services (including malaria control) for women and children; an initiative focused on evidence-based decision making to address leading causes of morbidity and mortality in the country. However there are variations in disease prevalence, health indices, health priorities and other socio-economic context[32, 33].

### Study design

This was a multi-phased mixed methods study[34]. Phase 1 included a baseline assessment workshop, where qualitative and quantitative data were collected from participants to identify their HPSR and GRIPP knowledge and capacity gaps. This was followed by three capacity building workshops. Phase 2 was the evaluation of the short term outputs of the capacity development intervention. The evaluation was undertaken three months after the last of the three capacity building workshops for the producers and users of evidence. The evaluation adopted a cross-sectional design, to assess implementation of HPSR and GRIPP activities proposed by respondents who had participated in a series of capacity building workshops in Anambra and Enugu states. A detailed description of the entire study intervention is outlined below to give adequate insight and background to the evaluation, but this paper reports only the findings of the quantitative component of the evaluation phase.

### Phase 1: Participant mapping, recruitment and workshops

Invited stakeholders included section heads of the Ministries of Health (MoH) with an emphasis on their Departments of Planning Research and Statistics (DPRS), and Departments of Public Health and Disease Control. For producers of research evidence, we invited heads of departments, core HPSR+A or HPSR-related senior researchers in the Public Health departments of the University of Nigeria Nsukka/Enugu-Campus, Enugu, Nigeria, Enugu state University, Enugu and Nnamdi Azikiwe University, Awka, Anambra state. In addition to strengthening capacity for control of NTDs and malaria, the researchers were also keen on a broader health system strengthening (HSS) and this was taken into consideration during the mapping of the users of evidence. Approvals were received from both states’ MoH for their staff to attend the workshops. Also, individual contacts were made by the research team to the different potential participants, to mobilise and sensitise them about the benefits of the project. An information sheet about the capacity building project was initially sent to identified stakeholders either physically or through e-mails. Explanatory invitation letters were physically dropped off in their offices. This was followed by phone calls and text messages, i) to confirm that letters/e-mails were received and ii) to check availability of the proposed participant for the baseline capacity assessment and subsequent workshops. We started by identifying all potential stakeholders and prioritised the invitations based on their position and area of work, i.e. prioritizing NTDs, malaria, MCH and stakeholders who were in a position to influence decisions that would impact on GRIPP. However, participants were also required to step-down the training to other relevant statekholders in their organizations, whom were then included in the evaluation. Workshops included an initial introductory and capacity needs assessment workshop and subsequently, three capacity building workshops (1,2 &3). After the initial braod introductory workshop, based on the resources available to the researchers, we invited a total of 60 participants (30 producers and 30 users) from Anambra and Enugu states for capacity building workshops 1 and 2, and for workshop 3, the number was increased to 120 (60 producers and 60 users) in both states. All initial participants for workshop 1 were also expected to attend workshop 2 and then a wider cohort was expected for workshop 3 (step down). Hence, participants who attended the third workshop consisted of some people who attended the first two workshops and some who did not attend either of the first two workshops. The actual attendance by participants is outlined in the Results section.

### Capacity needs assessment workshop

The baseline capacity assessment workshop was used to introduce the project to the participants face to face and assess their HPSR + A capacity needs. The needs assessment was conducted using a self-administered questionnaire. Questionnaires were also given to relevant stakeholders who could not attend the sensitization workshop. In-depth interviews on capacity gaps and needs were also conducted on a sub-set of participants, purposively selected based on their position in their organization or on their roles in various health programmes. Details of the process and findings from the quantitative and qualitative situational analysis have been published and are available as open access.[35, 36]

### Capacity building workshops

In workshops 1 and 2, we built the capacity of participants in HPSR+A and techniques (GRIPP). In the third workshop, we stepped down the training that was provided in the first and second workshops to a wider group. Each workshop was held for 2 days. The first and second workshops were a mix of producers and users of evidence in attendance. The third workshop consisted of one day of parallel sessions on strategies of GRIPP for producers and users of HPSR evidence. On the second day of the third workshop, a combined session for producers and users of evidence was held, during which participants were grouped into four thematic knowledge networks based on their previous area of work or interests, or their current work or interest. It was ensured that there was at least one top level manager/head of department in each thematic group. The thematic networks comprised both producers and users of evidence and are namely, NTDs, malaria, MCH, and HSS. In each workshop, the programme was scheduled to start with the lectures first, followed by interacive sessions for clarifications and then group work, with short breaks in between. During group works, participants were divided into either thematic or topic groups and allocated a faclitator from the research team and a moderator who could be a participant. At the end of group works, participants came back together in the larger group to feed back the group work outputs.

#### Workshop 1

Lectures were delivered on HPSR+A and Health Economics using power point presentations, followed by group work, to reinforce understanding using case studies. Prior to the lectures, a test of 15–20 multiple choice questions on each subject was administered to participants and their scores were recorded. After the lectures, the same questions were re-administered to check understanding. The HPSR topics were- Introduction to Health systems, the WHO Building Blocks, Introduction to Policy Analysis and Stakeholder Analysis. For Health Economics, the concepts of Demand and Supply, fundamentals of assessing Cost-Effectiveness and Basic Principles and Importance of Costing Health interventions were taught. These topics were all geared towards improving evidence-based health care decision making for policies and programmes.

#### Workshop 2

This workshop was used to introduce the concept of GRIPP. It focused on researcher-user engagement and different models of GRIPP that have worked for the evidence producers in the past. Strategies of communicating research findings were discussed with participants based on previous activities and experiences of the HPRG researchers which had produced positive results[37].

#### Workshop 3

The third workshop consisted of one day of parallel sessions for producers and users of evidence. Participants (producers and users) who had been trained in workshops 1 and 2 were now made trainers to step down the knowledge to a wider audience. On the second day of this third workshop, a combined session for producers and users of evidence was held, during which participants were grouped into four thematic knowledge networks (i; NTDs; ii; Malaria; iii; MCH; iv; HSS) based on their area of work in their organization or interests. It was ensured that there was at least one top level manager/head of department in each thematic group. In their various groups, they proposed activities they were going to carry out in their various organizations in the short term (three months), medium and long term, after the workshop. Although, three-month post intervention evaluation may be considered a short period, given other competing responsibilities of participants [36], a longer period would have fallen out of the project duration. Bearing this in mind, participants were advised to propose realistic activities that they could undertake within this time frame. They were also to set medium- and long-term goals.

### Phase 2: Evaluation of outputs from capacity building

#### Sampling and sample size

A total of 118 producers and users of research evidence who attended the workshops were surveyed. Respondents included researchers, academia, policy makers, programme/project managers, M&E officers, directors in health agencies and parastatals, members of civil society organizations and media representatives as earlier outlined.

#### Data collection

Two structured questionnaires (one for producers and the other for users) were used to collect information on participants’ progress with implementation of proposed short term HPSR+A activities and the effects of these activities on getting research evidence into policy and practice within their respective organizations, units or departments. The questionnaires were designed for this study using information on participants’ planned activities for improving evidence-based decision making for control of endemic tropical diseases. Hence, the questionnaires were developed by the researchers. following literature review and also drawing from a previous capacity needs assessment carried out by the researchers [38]. Information collected using the questionnaire include; which of the workshops attended by respondent; if identified activity had been initiated, completed, in progress, not done. Where activities had not been carried out, we also explored the reasons (No authority, Financial constraints, time constraints, lacked team support). The questionnaires were self-administered but research assistants were present to provide clarifications to participants. Questionnaires were retrieved on the same day they were distributed to respondents. The questionnaire was designed in a way that enabled the use of the expected outputs from the capacity development activities to be used to track outcomes. All participants were required to answer all questions and not restricted to only questions relating to their identified thematic group, hence options included ‘ Done’ ‘work in progress’ ‘Not done’ and ‘Don’t know’ However questionnaires were tailored to whether they were producers or users for each state, given that they had identified different activities. Hence there were four questionnaires. Because implementation was evaluated at the individual level, a “not done “response could mean that the activity was outside the participant’s group, e.g. a “not done” response could be given for an activity concerning NTD by a participant in the MCH group.

#### Data analysis

Date were entered and analysed with the aid of the Statistical Package for Social Sciences (SPSS) Version 16. The participants’ proposed activities for GRIPP, plans, and strategies in the control of endemic diseases were disaggregated by their thematic areas. Response to variables were summarised in percentages and presented separately for producers and users respectively. Because we are not comparing the two states, given that the activities proposed were different, both states are presented on the same table.

### Ethics

Ethical approval (NHREC/05/01/2008B-FWA00002458-IRB00002323) was obtained from the Health Research Ethics Committee of the University of Nigeria Teaching Hospital, Enugu, Enugu State, Nigeria. Written informed consent was obtained from respondents and all questionnaires were anonymised using respondent codes.

## Results

In the results section, we first present the socio-demographic characteristics of participants followed by what progress had been made with implementing identified activities in three months. We then present what GRIPP activities had been carried out as a result of implementing these activities or as a result of applying knowledge and insights gained from the capacity building workshops. We also present reasons given by participants for not implementing the tasks as proposed.

### Demographic characteristics of producers and users of evidence

All 118 questionnaires administered were retrieved and included in the analysis. These comprised 23 producers of evidence and 37 users of evidence in Anambra state, and 31 producers of evidence and 27 users of evidence in Enugu state. Table 1 shows the demographic characteristics of producers and users of evidence in both States. There was an almost equal gender distribution; while producers of evidence constituted 44.4%. Approximately three-quarters of respondents had spent less than 5 years in their current roles, 74.1% of producers of evidence and 76.6% of users of evidence. Other demographic characteristics are presented in the table below.

**Table 1:** Characteristics of study respondents.

Variables	Both states n (%)	Variables	Both states n (%)
**Producers of evidence (N = 54)**		**Users of evidence (N = 64)**	
**Gender**		**Gender**	
Male	29 (53.7)	Male	26(40.6)
Female	25 (46.3)	Female	38(59.4)
**Age group (years)**		**Age group (years)**	
25–40	19(35.2)	25–40	36(56.3)
41–50	26(48.1)	41–50	14(21.9)
51–60	7(12.9)	51–60	13(20.3)
>60	2(3.7)	-	-
**Main role in institution**		**Main role in organization**	
Department/Divisional head	4(7.4)	Director / head of department	24(37.5)
Consultant	8(14.8)	Program manager	8(12.5)
Lecturer/Researcher	24(44.4)	Implementers**	32(50.0)
PHD Student	5(9.3)		
Others (resident doctors/registrars)	13(24.1)		
**Highest educational level**		**Highest educational level**	
Higher National Diploma	1(1.9)	Higher National Diploma	4(7.8)
Bachelor’s degree	5(9.3)	Bachelor’s degree	36(56.3)
Master’s degree	25(46.3)	Master’s degree	17(26.6)
PhD/Fellowship	23(43.6)	PhD/Fellowship	2(3.1)
		Others e.g. Diploma	4(6.3)
**Time spent in current role (years)**		**Time spent in current role (years)**	
<5	40(74.1)	<5	49(76.6)
5–10	8(14.8)	5–10	15(23.4)
>10	6(11.1)	>10	0(0.0)
Capacity building workshop(s) attended*		Capacity building workshop(s) attended*	
Workshop-1 (HPSR & Health Economics)	23(43.6)	Workshop-1(HPSR & Health Economics)	24(37.5)
Workshop-2 (GRIPP)	19(35.2)	Workshop-2 (GRIPP)	24(37.5)
Workshop-3 (Step-down)	39(72.2)	Workshop-3 (Step-down)	48(75.5)
**Name of organization**		**Name of organization**	
COOUTH	2(3.7)	State Ministry of Health	44(68.8)
NAUTH	21(38.9)	SPHCDA	10(15.6)
ESUTH	9(16.7)	Implementing partner	1(1.6)
UNN	5(9.3)	SMEPB	6(9.4)
UNEC	10(18.5)	Hospital Management Board	1(1.6)
UNTH	7(12.9)	House of Assembly	1(1.2)
		State Ministry of Information	2(3.1)

COOUTH-Chukwuemeka Odumegwu Ojukwu University Teaching Hospital, Awka Anambra state; NAUTH-Nnamdi Azikiwe University Teaching Hospital Nnewi, Anambra state; ESUTH-Enugu State University Teaching Hospital, Parklane Enugu state; UNN-University of Nigeria, Nsukka Enugu state; UNEC-University of Nigeria, Enugu campus Enugu state; UNTH-University of Nigeria Teaching Hospital Ituku Ozalla, Enugu state; SPHCDA-State Primary Health Care Development Agency; SMEPB- State Ministry of Economic Planning & Budget.

* = Multiple responses allowed

**Includes planning officer, logistician, program analyst, nursing officer

***Social Work, Sociology and Anthropology

Attendance rate at all workshops was high as outlined in Table 2. While some attended all three workshops, some attended two and others only the step-down workshop.

**Table 2:** Attendance at Capacity building workshops by participants.

Training workshops organized by Health Policy Research Group	Number proposed/invited	Number of participants in each workshop		
	Producers and Users in both states	Producers of evidence (N = 54)	Users of evidence (N = 64)	Attendance rate (%)
Workshop 1: Training of trainers on Health Policy and Systems Research & Economic evaluation	60	23	24	78.3
Workshop 2: Training of trainers on Getting Research into Policy and Practice	60	19	24	73.3
Workshop 3: Step-down training on HPSR, Health Economics and GRIPP	120	39	48	72.5

*Some participants attended more than one workshop

### Short term activities proposed by participants

For their short-term goal activities, which were evaluated and reported in this study, participants identified a number of activities (Anambra producers = 10, Enugu Producers = 14; Anambra users = 17; Enugu users = 14). Given the relatively short interval (three months), participants were advised to realistically prioritize one or two activities per thematic group. It is those prioritized activities that we report below. Selected activities which participants identified as key, in their respective groups are outlined below but results of entire list of activities undertaken will be attached as additional tables. Although tasks were identified in groups, participants were encouraged to share updates across groups during the execution period as they were all in the same organization (users-State Ministry of Health & Producers-Academic Institutions). Based on this, evaluation questionnaires were also administered to individual participants and not as a group, consequently the unit of analysis was also on the participant (individual) level.

Table 3 shows that the users and producers of evidence suggested different and divergent activities that they will undertake in Anambra state. Whilst the producers of evidence focused mostly on activities on research, the users of evidence focused mostly on activities that will improve the implementation of health plans. However, the users of evidence also suggested some research-related activities for malaria and MCH services.

**Table 3:** List of activities proposed by producers and users of evidence in Anambra State.

Anambra State	
Producers	Users
**Neglected Tropical Diseases (NTDs)**	
Literature review to update data on prevalence of NTDs	Identify LGAs mostly affected by NTDs, endemicity in those areas and drug distribution in 2016
Undertake advocacy/networking with Ministry of Health for evidence-based decision making in NTDs	Build capacity of other stakeholders/health workers in NTD control on evidence informed decision making (step down training)
**Malaria**	
Initiate steps to implement survey on availability of malaria diagnostic tools and personnel	Identify research priorities for malaria
Establish knowledge network with users of evidence Anambra state for malaria control	
**Maternal and Child Health (MCH)**	
Initiate steps to implement study on immunization uptake by pregnant women	Initiate assessment of uptake of maternal health care services
Sensitize stakeholders in MCH on evidence-based decision making	Conduct an assessment of childhood malnutrition, malaria and other illness
**Health system strengthening (HSS)**	
Organize training workshops for health workers in NAUTH on prompt patient care to reduce hospital waiting time	Undertake stakeholder sensitization on evidence informed decision making

Table 4 shows that users of evidence in Enugu state proposed amongst other things to further train their colleagues that were not part of the workshops. The users of evidence in Enugu also proposed some operations research activities on NTDs, Malaria control, MCH and HSS. A major activity that was proposed by the producers of evidence in Enugu state was the formation of a research network for the evaluation of service delivery in the state.

**Table 4:** List of activities proposed by producers and users of evidence in Enugu State.

Enugu State	
Producers	Users
**NTDs**	
Identify laboratories for confirmatory diagnosis of NTDs in the state	Review of data and activities of community directed distributors
**Malaria**	
Undertake SWOT analysis of current malaria program in the state	Step-down of HPSR modules to malaria programme staff in SMoH
	Evaluate distribution of LLINs in Enugu State
**MCH**	
Assess training needs of personnel for collection of maternal and child health data	Introduce periodic survey in collaboration with management unit of Free MCH programme
	Advocacy visits to women groups for improved MCH decision making
**HSS**	
Form a research network to evaluate health services delivery	Capacity building and sensitization of colleagues on evidence-informed decision making in organization

### Implementation of proposed key activities among producers of evidence

Results of implementation of activities identified as key, by producers of evidence in both states are presented below (Table 5). Anambra state identified seven activities while Enugu state identified five. These were activities they prioritised out of other selected activities to carry out in the first three months after the capacity building workshops and participants reported in the questionnaires whether the activity had been carried out or not. In Anambra state almost 40% of respondents reported that Literature review to update data on NTDs and advocacy to the Ministry of health for evidence-based decision making for control of NTDs had been carried out respectively. Steps had also been taken to advocate evidence-based decision making to MCH stakeholders (17.4%) and surveying malaria diagnostic tools (17.4%). The results show that most activities were still in progress and some had not been carried out as expected. In Enugu state, just like in Anambra, activities identified for better control of NTDs were the ones most carried out.

**Table 5:** Results of implementation of key activities identified by producers in both states.

Activity	Done	Not done	In progress	Don’t know	Total
**Anambra State (n = 23)**					
Literature review to update data on prevalence of NTDs (n = 23)	9(39.1)	8(34.8)	4(17.4)	2(8.7)	23(100.0)
Undertake advocacy/networking with Ministry of Health for evidence-based decision making in NTDs	9(39.1)	9(39.1)	3 (13.0)	2(8.7)	23(100.0)
Initiate steps to implement survey on availability of malaria diagnostic tools and personnel	4(17.4)	12(52.2)	2(8.7)	5(21.7)	23(100.0)
Establish knowledge network with users of evidence Anambra state for malaria control	3(13.0)	11(47.8)	3(13.0)	6(26.1)	23(100.0)
Initiate steps to implement study on immunization uptake by pregnant women	4(17.4)	14 (60.9)	1(4.3)	4 (17.4)	23(100.0)
Sensitize stakeholders in MCH on evidence-based decision making	4(17.4)	12(52.2)	3(13.0)	4(17.4)	23(100.0)
Organize training workshops for health workers in NAUTH on prompt patient care to reduce hospital waiting time	1 (4.3)	17 (73.9)	5 (21.7)	0	23(100.0)
**Enugu State (n = 31)**					
Identify laboratories for confirmatory diagnosis of NTDs in the state	10 (32.3)	19 (61.3)	2 (6.5)	0	31 (100)
Create awareness of NTD laboratories among health workers in the State	8(25.8)	18(58.1)	2(6.5)	3(9.7)	31 (100)
Undertake SWOT analysis of current malaria program in the state	3 (9.7)	22 (71.0)	5 (16.1)	1 (3.2)	31 (100)
Assess training needs of personnel for collection of maternal and child health data	2 (6.5)	23 (74.2)	4 (12.9)	2 (6.5)	31 (100)
Form a research network to evaluate health services delivery	8 (25.8)	14 (45.2)	7 (22.6)	2 (6.5)	31 (100)

### Implementation of proposed activities by users of evidence in both states

In Anambra state, the activity where the most progress had been made was undertaking stakeholder sensitization on evidence informed decision making (32.4%) and step down capacity building of other colleagues in their organization on evidence informed decision making (29.7%). Advocacy visits to women groups for improved MCH decision making was the key activity that been most implemented by users of evidence in Enugu state. Progress with other activities are shown in Table 6. While the producers mainly reported that most of the activities were in progress, majority users, especially in Anambra state did not have knowledge of identified activities.

**Table 6:** Results of implementation of key activities identified by users in both states.

Activity	Done	Not done	In progress	Don’t know	Total
**Anambra State (n = 37)**					
Identify LGAs mostly affected by NTDs, endemicity in those areas and drug distribution in 2016	6 (16.2)	3 (8.1)	3 (8.1)	25 (67.6)	37(100.0)
Build capacity of other stakeholders/health workers in NTD control on evidence informed decision making	3 (8.1)	5 (13.5)	3 (8.1)	26 (70.3)	37(100.0)
Identify research priorities for malaria	4 (10.8)	6 (16.2)	3 (8.1)	24 (64.9)	37(100.0)
Initiate assessment of uptake of maternal health care services	2(5.4)	13 (35.1)	3(8.1)	19(51.4)	37(100.0)
Conduct an assessment of childhood malnutrition, malaria and other illness	4(10.8)	9 (24.3)	1(2.7)	23(62.2)	37(100.0)
Undertake stakeholder sensitization on evidence informed decision making	12(32.4)	6 (16.2)	4(10.8)	15(40.5)	37(100.0)
Capacity building and sensitization of colleagues on evidence-informed decision making in organization	11(29.7)	8 (21.6)	4(10.8)	14(37.8)	37(100.0)
**Enugu State (n = 27)**					
Review of data and activities of community directed distributors	5 (18.5)	15 (55.6)	0	7 (25.9)	27(100)
Step-down of HPSR modules to malaria programme staff in SMOH	4 (14.8)	22 (81.5)	1 (3.7)	0	27 (100)
Evaluate distribution of LLINs in Enugu State	4 (14.8)	10 (37.0)	4 (14.8)	9 (33.3)	27 (100)
Introduce periodic survey in collaboration with management unit of Free MCH programme	3 (11.1)	8 (29.6)	9 (33.3)	7 (25.9)	27 (100)
Advocacy visits to women groups for improved MCH decision making	9 (33.3)	13 (7.3)	1 (3.7)	4 (14.8)	27 (100.0)

### Influence on GRIPP as a result of implementation of proposed activities in Anambra and Enugu state

Tables 7 and 8 show the influence of implementation of proposed activities on GRIPP, by producers and users of evidence in both States. In Anambra state, 26.1% of producers of evidence reported that their activities had influenced modification of existing policies, programme plans or strategies and M&E frameworks; and 47.8% reported that implementation of proposed activities had influenced research priority setting in their organization. Among users of evidence in the State, ten people (27%) reported that advocacy visits were paid to the commissioner and permanent secretary of health to deliberate on the outcome of the workshop. Only one person reported that research evidence had influenced decisions in childhood immunization and illnesses such as malaria and diarrhoea.

**Table 7:** GRIPP Outcomes of activities carried out by producers in both states.

**Anambra**	**N = 23**
Modification of existing policies, programme plans or strategies for control of endemic diseases in the State	6.(26.1)
Modification of Monitoring and Evaluation frameworks in the State	6.(26.1)
Research priority setting in organization	11(47.8)
**Enugu**	**N = 31**
Disseminated information on malaria control program policy makers, and other stakeholders	3(9.7)
Disseminated findings on human resource for MCH services to stakeholders and public	1(3.2)
Shared policy briefs on MCH findings with key stakeholders in the State	1(3.2)

**Table 8:** GRIPP Outcomes by Users in both states.

**Anambra**	**N = 37**
Used evidence from assessment of childhood malnutrition and illnesses to influence decisions in malaria control, immunization and management diarrhoea	1(2.7)
Paid advocacy visit to the commissioner and permanent secretary of health to deliberate on the outcome of the workshop	10(27.0)
**Enugu**	**N = 27**
Advocacy to decision makers in health, budget and planning and local government	4(14.8)
Involvement in knowledge translation, use and management with malaria unit	6 (22.2)
Knowledge network on free MCH	6(22.2)
Interactive sessions with the Health Policy Research Group	8(29.6)
Influenced existing health policies, programme plans or strategies	9(33.3)
Influenced modification of M&E frameworks in the State	10(37.0)
Influenced research priority setting in organization	8(29.6)

GRIPP outcomes of implementation of proposed activities were more pronounced among users of evidence in Enugu state compared to producers of evidence; 9.7% of producers of evidence reported they had disseminated information on malaria control program to policymakers, and 3.7% each reported they had disseminated findings on human resource for MCH services to key stakeholders through policy briefs. Among users of evidence in the State, 14.8% stated that advocacy had been paid to decision makers in health and other stakeholders; 22.2% said they had been involved in knowledge translation and use and management within malaria control unit and an equal proportion said they were involved in knowledge network for free MCH; 29.6% reported that they had interacted with HPRG formally since after the workshop. With respect to modification of policies and plans, 33.3%of users of evidence in the State reported that their activities resulted in modifications to health policies, plans and strategies in malaria control, NTD, MCH and health systems strengthening; 37% reported that activities had resulted in modification of M&E frameworks; and 29.6% said the activities had influenced research priority setting in their organization. A particular thematic network that was formed during the workshops, “Maternal and Child Health (MCH) network” was able to launch a social media platform, which has enabled the Enugu Ministry of Health as an organization to be socially visible and get feedback from external customers, with the simultaneous enablement for internal communication between staff.

### Reasons for not implementing proposed activities (external and internal) to influence GRIPP

Respondents were also asked for reasons for not having implemented proposed activities, where this was the case. The options were given in the questionnaire were i) time constraints; ii) financial constraints; iii) Lack of authority to implement activity; iv) Lack of support from team; v) Loss of interest in proposed activity after the capacity building workshop; vi) Other. Multiple responses were allowed. Whilst lack of authority to implement was the most common reason for all activities proposed by Anambra producers, most common reasons amongst Enugu producers were more varied, but with lack of authority still being most common reasons shown in Table 9. Amongst users of evidence, in both states, financial constraint was the major reason for not implementing proposed activities in Anambra state, while in Enugu state it was lack of authority. (Table 9). This was closely followed by lack of authority to implement, and then time constraints a distant third. However, these were not tested for statistical significance.

**Table 9:** Summary of Most common reason for not implementing activities amongst participants in both states.

Producers			
**Anambra**		**Enugu**	
**Total No of Activities**	**Most common reason (%)**	**Total No of Activities**	**Most common reason (%)**
10	No authority (100.0)	14	No authority (42.9)
**Users**			
**Total No of Activities**	**Most common reason (%)**	**Total No of Activities**	**Most common reason (%)**
17	Financial constraints (70.6)	14	No authority (71.4)

## Discussion

This study looked at whether building the capacity of relevant stakeholders can lead to enhanced evidence-based decision making for better control of endemic diseases in Nigeria. The results highlight three main findings i) producer-user engagement which resulted in participants undertaking to change their practice within a short time (3 months); ii) Implementation of activities as identified despite constraints iii) Modification of practice towards improving control of endemic diseases and overall health system strengthening. Reasons for not engaging in proposed activities were also highlighted and these were also qualitatively explored in the larger study of which this is a component.

A variety of producers and users of evidence, as seen from the socio-demographics actively participated in the capacity building workshops. This engagement afforded an opportunity to reinforce producer-user dialogue, the absence of which is known in the literature to constrain GRIPP. Weak linkages between producers and users of evidence have been reported severally in literature[37–39] as a constraint to knowledge translation, and we also found this to be a factor in our study context as reported in the baseline assessment[36]. Studies have shown that when producers of evidence engage end users early enough in the evidence production process, that it facilitates decision making in policy and practice[37, 40] In 2003, a study showed strategies to improve stakeholder engagement in LMICs were either not undertaken, or where they were undertaken, were not successful[6], so successfully carrying out this producer-user engagement servers to improve on the necessary task of building a critical mass of producers and users of HPSR evidence for the control of endemic diseases, and for overall health system strengthening in LMICs. These engagements also foster a sense of collective ownership of the evidence and hence smoother knowledge translation[41].

Participants committed and took initiatives to implement activities to change or improve their practices and were reported to have already implemented some activities, while some others were in progress, in the short period of three months following the capacity building exercise. In the last two decades a number of strategies have progressively encouraged these engagements in different country contexts as reported in a recent summary of the progress of HPSR. These were in form of events or publications[42].

Participants were able to influence some GRIPP activities in their organization by applying new skills learnt during the capacity building. As a result of new skills and competencies, participants reported successes in influencing a number of GRIPP activities in both states, namely, revising programme plans and existing M&E frameworks, and also incorporating evidence for better malaria control. These are very encouraging steps towards evidence-based practice, especially as this was just within three months of capacity building. Secondly, this covertly also implies a changing mindset which is key to changing practice, and begins to challenge resistance to change, which has been reported as one of the constraints to achieving GRIPP, amongst others[37].

The study also throws up a number of constraints to achieving these goals in the study contexts. Of the various reasons given by participants for not implementing proposed activities, lack of authority was the major reason amongst producers of evidence, in both states and amongst users of evidence in Enugu state. Lack of authority reported by users, is a concern, because it was felt that, given the higher position of some of the participants, they would be able to implement some proposed changes, even if not all, and for this reason, were purposively allocated to be present in each thematic group during the capacity building workshop. Although, these were not tested for statistical significance because of small sample sizes, the finding forms an initial step to interrogating an implied narrow or non-existent decision space, which is being explored in the qualitative component of this project[36].

Financial constraint was the second most common reason for not implementing proposed activities, while time constraint was the third common reason for not applying evidence from HPSR for decision making. Time constraint may be as a result of the short duration (3 months) between capacity building and evaluation. However, this may also be due to participants not having yet changed mindsets adequately to prioritise these activities, given that there will always be competing activities and deadlines from other activities. Financial constraints along with time constraints have also been reported in previous studies in Nigeria and other LMICs [20, 37, 43, 44]. Over a decade ago, in 2004, a summit on HPSR called for increased funding for HPSR in LMICs; and that call appears to still remain pertinent presently[10].

Another study carried out in a different state in Nigeria recognises that strategies as employed in our study have been shown to improve evidence-based policy making and implementation in developed countries and are likely to produce better outcomes in developing countries like Nigeria, if these constraints are addressed[24]. The study also suggests a supply driven approach to capacity strengthening initiatives based on the assumption that if the skills of the main actors- (researchers and policy makers)-are enhanced via trainings and enough institutional capacity is built, these will then tend to put research outputs to good use [24].

These findings from evaluation of participants’ short-term goals reveal that they will require further support in completing their short-term goals, since some of the activities were still in progress, and also for carrying out their medium- and long- term goals. The main cross cutting constraints to the use of evidence from HPSR for decision making were limited decision space/authority to make GRIPP changes, time and funding. Continued support is needed for longevity and sustainability, in order to realise the HPSR mandate of building a critical mass in LMICs [2, 45].

Finally, for sustaining GRIPP, mainstreaming HPSR as a subject into under- and post-graduate teaching in developing countries, building a collective of emerging leaders in HPSR and aligning HPSR capacity strengthening within the wider organizational development are also key strategies to be embarked upon, going forward [30, 38]. A concept of establishing multi-stakeholder research triads consisting of researchers, community members and policy makers to jointly establish local health research agenda needs to be explored, as this will foster knowledge translation when research evidence is made available [46]. Community members and policymakers also need to be embedded in research teams and undertake research projects, as this will facilitate knowledge translation[47]. There is also an imperative for advocacy for sustained sensitization and support for stakeholders whose capacities in HPSR have been built to be involved in decision-making, with continued expansion of the mass of producers and users of HPSR evidence with the requisite knowledge in HPSR and GRIPP and building of networks between producers and users of evidence.

The strengths of this study are that it not only created the awareness on the knowledge and capacity gaps for HPSR production and use, but also embarked on filling identified gaps. The previous experience of the researchers in HPSR and the already established linkages and trust that the team has with most of the decision makers (research users) also facilitated the project.

A limitation of the study was the short duration between the implementation of the interventions and the evaluation of their effects in terms of the participants’ goals. This was done in order to include at least one evaluation within the project period which was just 12 months. Studies have shown that further support is necessary for achievement of set goals, especially the long term ones [20, 48]. It also appears, in retrospect, that too many activities were proposed for a three-month period and may not have been realistic. Other limitations include the fact that only two domains of capacity (skills improvement and interaction/networking for GRIPP) were targeted in this intervention. There is need in future to expand to building organizational and environmental/ network capacity to enhance resilience and sustainability of HPSR activities [20, 45, 48]. Area for future implementation research should include a longer implementation period which allows for a longer period of evaluation and re-evaluation with progressive support and mentoring and the use of an implementation framework which would aid future comparability[49]. Another limitation of this study was not establishing associations through statistical testing of significance, this should be incorporated in future evaluations.

## Conclusion

This study highlighted key findings from a 12 month implementation research project which built capacity of particpants. The key messages are that producers and users of HPSR evidence should engage each other at every opportunity. Getting research evidence into policy and practice can be initiated by making little changes in everyday practices of all stakeholders. Outstanding challenges to these activities, especially decision spaces, time and financial constraints need to be actively addressed, moving forward, not just for improving control of endemic diseases but towards strengthening the overall health system. Undertaking the study in two states helped to show the divergent and convergent outputs from two states that are neighbours and that had the same capacity development activity. Although, there was no direct comparison, it helps to illustrate the need for context-specificity in planning and undertaking activities.
